# Survey Evaluation of the Role of Social Media and Social Support for Transgender, Nonbinary, and Intersex People: Observational Study

**DOI:** 10.2196/79614

**Published:** 2026-01-13

**Authors:** Krishnan Nayar, Vivian C Iloabuchi, Juliana M Kling, Bithika Thompson, Christopher Dodoo, Robert Horsley

**Affiliations:** 1 Alix School of Medicine, Mayo Clinic in Arizona, Scottsdale, AZ, United States; 2 Division of Women's Health Internal Medicine, Mayo Clinic in Arizona, Scottsdale, AZ, United States; 3 Division of Endocrinology, Mayo Clinic in Arizona, Scottsdale, AZ, United States; 4 Quantitative Health Science Research, Mayo Clinic in Arizona, Phoenix, AZ, United States; 5 Community Internal Medicine, Mayo Clinic in Arizona, 13400 E Shea Blvd, Scottsdale, AZ, United States, 1 4803018087

**Keywords:** transgender, gender diverse, social support, social media, support group

## Abstract

**Background:**

Transgender and gender-diverse (TGD) people experience greater health disparities than their cisgender counterparts. Social determinants of health are linked to these health disparities in minority communities, including the TGD community. Lack of social support contributes significantly to these disparities for the TGD community.

**Objective:**

The aim of this study was to evaluate the role of social media and social support groups among TGD patients who attend a transgender clinic.

**Methods:**

A questionnaire was developed through an iterative process and emailed to TGD people attending a tertiary care TGD-focused clinic. The survey assessed social media use (platforms, duration, and adverse effects), social support groups (past participation and interest in current participation), and demographic characteristics (age, gender, race and ethnicity, educational level, religious affiliation, and income).

**Results:**

Our survey garnered 48 responses. Of these participants, 50% (n=24) identified as transfeminine or transgender women, 29.2% (n=14) identified as transmasculine or transgender men, 8.3% (n=4) identified as nonbinary, 2.1% (n=1) identified as genderfluid, and 10.4% (n=5) identified as another identity. Our respondents’ average age was 35 (SD 15.6) years. Nearly 70% (n=31, 64.6%) reported at least monthly transphobia, and 35.4% (n=17) reported at least weekly transphobia. Primary social support was reported as coming from an in-person significant other or friend 49% (n=24) of the time and from social media or online friends 12.5% (n=6) of the time. Social media was used for the primary purpose of interacting with queer or TGD people by 65% (n=33) of respondents, and the most common sites used were Discord, Reddit, and Instagram. Among respondents who either were attending or had attended a gender identity–focused support group, 61% (14/23) reported them being beneficial. In total, 52% (25/48) had never attended a support group related to their gender identity, and 60% (15/23) were open to attending.

**Conclusions:**

This study found that social media is already being used by TGD people for the purpose of interacting with other queer and transgender people but also that there are risks associated with its use. Given this reality, counseling patients on social media use should focus on safety in use and honest discussions of both the risks and benefits associated with its use. Regarding social support groups focused on gender identity, many current or previous attendants reported that support groups were helpful for finding social support, especially early on in one’s transition and when other avenues of support are not present. Additionally, many respondents who had never attended a support group were interested in attending for the perceived benefits of increased social support and interest in meeting other community members. Engaging TGD patients in the use of social media and social support groups for gender identity may help improve support, although exposure to hate and transphobia is a risk that comes with social media use.

## Introduction

Transgender and gender-diverse (TGD) people face hostile social and political landscapes in the United States. This includes rejection from social groups and family as well as discrimination both socially and politically [1]. Importantly, in relation to recent increases in antitransgender legislation, TGD people who have reported worry about rights being taken away have been shown to have higher rates of depressive and anxious symptoms [2,3]. Transgender people also face discrimination and harassment to a greater degree than cisgender people, including harassment specifically due to gender identity [4,5].

Largely as a result of discrimination and social stigma, TGD people experience greater health disparities than their cisgender counterparts. TGD people have higher rates of depression, attempted suicide, HIV, and experienced trauma. Nearly 60% of surveyed TGD people who reported a suicide attempt said that their family chose not to speak to or spend time with them or reported discrimination, harassment, or bullying at school and/or work [6].

The environments in which we live, work, and age can influence our health. Research has demonstrated correlations between social determinants of health and many health outcomes [7]. Social support is an important determinant of health [8]. An important component of social support, especially among younger populations, is social media. Social media has been shown to have both positive and negative effects on the mental health of queer people [9-11] but is understudied among TGD people of all ages. Moreover, tools used to measure social support are often underdeveloped when studying this population [12].

The objective of this study was to assess social media use among TGD people, including preferred platforms, reasons for social media use, and noted adverse effects associated with social media. Additionally, we aimed to gain a deeper understanding of the desires of TGD patients in relation to organized support groups.

## Methods

### Ethical Considerations

This study was reviewed and determined to be exempt from institutional review board approval (application 24-001890) by the Mayo Clinic institutional review board. This included a review of the finalized survey, recruitment email, and recruitment process as outlined in the application. Although this research focused on a minority population, this study was found to be exempt due to the lack of identifying information collected. Informed consent was collected in the form of a recruitment email that emphasized the optional nature of the survey, the benefits of completing the survey, and the fact that no known risks were identified in association with completing the survey. No identifiable information was recorded during the survey, and responses were recorded using HIPAA (Health Insurance Portability and Accountability Act)-compliant software. The participants were not compensated.

### Setting, Population, and Data Collection

The questionnaire was developed by the survey team through an iterative process as no validated questionnaire existed (see the adherence to the Checklist for Reporting Results of Internet E-Surveys protocol [13] in Checklist 1). Survey questions collected information about social media use, social support groups, and demographic characteristics. Social media use information ranged from sites used, hours used, and adverse effects noted. Social support questions gathered information on support groups centered on past participation and desire for present participation. Demographic information collected included age, gender, race and ethnicity, educational level, religious affiliation, and income. No other identifiable information was collected. Volunteers were recruited via email through the listserv of the lesbian, gay, bisexual, transgender, and queer employee resource group to review the questionnaire and provide feedback to the survey team. We had 3 volunteers who provided feedback on the questions and answer choices. The full survey is available in Multimedia Appendix 1.

TGD people receiving care from the Transgender and Intersex Specialty Care Clinic (TISCC) at the Mayo Clinic in Arizona were recruited for this study. There were no exclusion criteria among this population. A list of emails was compiled from patients who were seen at the TISCC at the Mayo Clinic in Arizona between August 16, 2021, and August 14, 2024. The list of emails was screened, and duplicates were removed. Emails were sent out with individualized links, which only allowed for 1 response for each individual.

Using this list, surveys with informed consent documentation were sent out via email with reminders sent at 10 and 20 days after initial survey distribution. Recruitment and survey completion took place over the months of September 2024 to October 2024.

### Statistical Analysis

Continuous variables were described using means, SDs, medians, IQRs, and ranges. Categorical variables were described using frequencies and proportions. Group comparisons were conducted using the Kruskal-Wallis test and chi-square test for continuous and categorical variables, respectively. All analyses were carried out using SAS (version 9.4; SAS Institute).

## Results

### Overview

A total of 48 responses, out of 236, were received, yielding a response rate of 20%. Additional demographics can be found in Table 1.

**Table 1.** Demographics of the survey respondents (N=48).

Characteristic	Values
Age (year), mean (SD)	35.5 (15.6)
Age (years), median (IQR; range)	28.5 (23.5-45.5; 18-74)
Gender identity, n (%)	
Transfeminine or transgender woman	24 (50)
Transmasculine or transgender man	14 (29.2)
Nonbinary	4 (8.3)
Genderfluid	1 (2.1)
Other	5 (10.4)
Educational level, n (%)	
Some high school	1 (2.1)
High school graduate or GED^a^	4 (8.3)
Some college or 2-year college degree	24 (50)
4-year college graduate	8 (16.7)
Higher than 4-year college degree	11 (22.9)
Race, n (%)	
White	41 (85.4)
Other or preferred not to answer	7 (14.6)
Employment status, n (%)	
Working full time	22 (45.8)
Working part time	9 (18.8)
Student	8 (16.7)
Not currently working	7 (14.6)
Retired	2 (4.2)
Household income (US $), n (%)	
<25,000	6 (12.5)
25,000 to 39,999	5 (10.4)
40,000 to 54,999	4 (8.3)
55,000 to 69,999	2 (4.2)
70,000 to 84,999	2 (4.2)
85,000 to 99,999	3 (6.3)
100,000 to 149,999	5 (10.4)
≥150,000	12 (25)
Preferred not to answer	9 (18.8)
Religious affiliation, n (%)	
Agnostic	15 (31.3)
Atheist	11 (22.9)
Mormon	1 (2.1)
Catholic	2 (4.2)
Other Christian	5 (10.4)
Judaism	1 (2.1)
Other	7 (14.6)
Preferred not to answer	6 (12.5)

a GED: General Educational Development.

### Social Support and Social Media Use of Our Population

A total of 50% (24/48) of the respondents indicated that their support came from a significant other, spouse, partner, or in-person friends as evidenced in Table 2. In total, 12.5% (6/48) of the respondents reported that their primary source of support regarding their gender identity was online friends or social media.

**Table 2.** Social support (primary resources and discrimination) and social media use (sites used and adverse effects; N=48).

Question	Participants, n (%)
“What is your primary resource for emotional support regarding your gender identity?”	
Spouse, partner, or significant other	14 (29.2)
Friends, in person	10 (20.8)
Therapy	8 (16.7)
Family	6 (12.5)
Friends, online	4 (8.3)
Social media	2 (4.2)
Support group	1 (2.1)
Other	3 (6.3)
“How often do you deal with transphobia in your daily life?”	
Never	16 (33.3)
Monthly	14 (29.2)
Daily	9 (18.8)
Weekly	8 (16.7)
Social media as social support—respondents who reported that their primary purpose for using the following sites was “Interaction with other queer, transgender, or gender diverse people”:	
Discord	18 (37.5)
Reddit	9 (18.8)
Instagram	6 (12.5)
Tumblr	5 (10.4)
TikTok	4 (8.3)
Snapchat	3 (6.3)
X (formerly known as Twitter)	3 (6.3)
Facebook	2 (4.2)
YouTube	1 (2.1)
LinkedIn	0 (0)
Pinterest	0 (0)
“Do you struggle with any of the following in relation to social media?”	
Exposure to hate	27 (56.3)
Exposure to transphobia	26 (54.2)
Self-consciousness	22 (45.8)
Overuse	16 (33.3)
Bullying	3 (6.3)
Addiction	1 (2.1)
Other	7 (14.6)
Did not use social media	2 (4.2)

Regarding social media use as support for gender identity, 64.6% (31/48) of the respondents reported that they used at least one social media platform with the primary purpose of interacting with other queer or TGD people. The most common social media sites used with this as the primary purpose were Discord, Reddit, and Instagram, in that order.

Nearly 70% of respondents (31/48, 64.6%) reported experiencing transphobia at least monthly, with 35.4% (17/48) reporting experiencing transphobia at least weekly. Nearly 60% of the respondents reported that they were exposed to hate and/or transphobia on social media (27/48, 56.3% and 26/48, 54.2%, respectively), with these being the most common adverse experiences reported in relation to social media. Other common reported adverse experiences included self-consciousness and overuse.

### Social Support Group Desires and Attitudes

In free-text responses, participants mentioned that social support groups helped allow attendees to “meet others like me” and that they helped in “extending my support network.” Other responses mentioned the importance of social support groups, especially after recently coming out.

Among the 14 participants who reported that they had participated in a social support group but were no longer attending, responses were mixed about the effectiveness of support groups (Table 3). Some respondents reported that they were helpful, stating that “community is important when I first came out” and “social support groups are helpful by providing people with a safe space to exist.” However, other respondents reported that they were less helpful, citing “differences” between themselves and other participants. Of these 14 respondents, approximately half (7/14, 50%) reported that they either were or were maybe interested in attending a social support group once again. Responses referenced the importance of making friends and social connections, whereas others stated the difficulties of making time for such commitments.

**Table 3.** Social support groups: use, perceived benefits, and desire for attendance.

	Participants, n/N (%)
“Are you currently, or have you ever attended a support group?”	
“Yes, and I’m currently attending”	9/48 (18.8)
“Yes, but no longer attending”	14/48 (29.2)
“No”	25/48 (52.1)
“Was this beneficial to you?” (Among responses of “Yes, and I’m currently attending”)	
Yes	8/9 (88.9)
No	1/9 (11.1)
“Was this beneficial to you?” (Among responses of “Yes, but no longer attending”)	
Yes	6/14 (42.9)
Mixed	2/14 (14.3)
No	6/14 (42.9)
“Would you like to attend a support group?” (Among responses of “Yes, but no longer attending”)	
Yes	5/14 (35.7)
Maybe	2/14 (14.3)
No	7/14 (50)
“Would you like to attend a support group?” (Among responses of “No”)	
Yes	9/25 (36)
Maybe	6/25 (24)
No	10/25 (40)

In total, from the population of respondents who were not currently attending a support group, 56.4% (22/39) reported that they either were interested or were maybe interested in attending a social support group. Respondents who were interested in attending a group cited wanting more “social interaction,” “community,” and “support.” Some respondents who were not interested in attending a social support group reported that their support systems were “enough” and “adequate.”

## Discussion

### Principal Findings

In a group of TGD people presenting to a tertiary care clinic, most (33/48, 65%) used social media to connect with people in their community. However, a smaller percentage (2/48, 4.2%) turned to social media as their primary source of social support. Among those who used social media, Reddit, Discord, and Instagram were the most popular social media sites used by respondents specifically for the purpose of interacting with other queer and transgender people. Our survey also demonstrated concerning risks, including exposure to hate, transphobia, and self-consciousness in association with social media use, but also benefits in finding community and support.

Beyond the use of social media, our results demonstrated that individuals such as spouses, friends, and family are important for social support. In our study, many of those who were attending a social support group reported that they found participation to be beneficial, especially for social support and for finding others in the community like themselves. Among those who had attended a social support group but did not find it beneficial, a common theme was differences between other participants and themselves. Among respondents who had never attended a support group, many reported some level of interest in attending a support group and cited interest in finding community, support, and others like themselves. Among those who did not report interest in attending support groups, many reported already having enough support among family and friends or that they were concerned about differences (including demographic differences such as age and years since transition began) between potential support group participants and themselves. This points to social support groups as a potential valuable resource, especially for those without other avenues of support.

### Comparison With Past Literature

Prior studies have demonstrated the importance of social media as a tool for social support among TGD populations, although more research on social media use as support for TGD individuals has been focused on youth populations [14]. Social media has been shown to facilitate transgender adolescents’ connections with peers and identification with role models. Among lesbian, gay, bisexual, transgender, and queer adults, social media has been identified as a place to seek social support from other individuals who share facets of one’s identity (such as sexual orientation and race) [15,16]. The use of social media among younger populations has been thoroughly studied and shown to have effects such as exposure to cyberbullying and impacts on mental health [17]. Past studies have shown that, among TGD adults, exposure to transphobia, cyberbullying, and victimization has been noted as a potential adverse effect of social media use [18,19]. Beyond social media and known friends and family, in-person support groups have been found to be beneficial for marginalized communities [20] and specifically TGD people [21]. This is consistent with other studies [22] that have shown that TGD people who have never attended a support group view them as ways to find peer support and community. Social workers can engage patients and their families in the forming of these support groups and look to well-established clinics for resources [23].

### Limitations

The limitations of this study include the small sample size as well as the lack of an existing validated questionnaire. As no validated questionnaire exists specifically for the purpose of assessing social support among TGD people in relation to gender identity, we had to create our own. Additionally, our survey response rate was 20% (48/236). A systematic review found that the average response rate in counseling journals is approximately 34.2% [24], which puts our response rate slightly below but within 1 SD of the average from the latter study. Still, this relatively low response rate could introduce nonresponse bias, and to better understand our population, we will need a future study with a strategy for a higher response rate. Finally, our sample for this study comprised patients at the TISCC at the Mayo Clinic in Arizona, which is not demographically representative of the TGD community across the country. Thus, our survey suffers from nonresponse bias and, additionally, may not be generalizable to transgender adults across the United States.

### Conclusions

Our data help further the understanding of social media use and social support groups for TGD people to better inform physical and mental health professionals as to how to best meet the needs of TGD people. This study found that social media is already being used by TGD people for the purpose of interacting with other queer and transgender people, but also that there are risks associated with its use. With both positive and negative effects of social media use among TGD adults, counseling with patients seeking social support can be focused on safety in use. Although more research on this is necessary, forums where interactions occur among known groups with moderators (such as Discord and Reddit, identified by survey respondents as being used for connecting with other queer individuals) may provide safer ways to find social support.

In addition to social media, support groups were reported by many attendees to be helpful for finding social support, especially early on in one’s transition and when other avenues of support are not present. Offering support groups as part of TGD care may be something to consider given the disproportionate social stigma and resultant health disparities faced by this population.

## Supplementary material

10.2196/79614 Multimedia Appendix 1 Survey as presented to participants.

10.2196/79614 Checklist 1 CHERRIES checklist.

## Abbreviations

HIPAA: Health Insurance Portability and Accountability Act
TGD: transgender and gender-diverse
TISCC: Transgender and Intersex Specialty Care Clinic

## References

[1] White Hughto JM, Reisner SL, Pachankis JE (2015). Transgender stigma and health: a critical review of stigma determinants, mechanisms, and interventions. Soc Sci Med.

[2] Restar A, Layland EK, Hughes L (2024). Antitrans policy environment and depression and anxiety symptoms in transgender and nonbinary adults. JAMA Netw Open.

[3] Last BS, Tran NK, Lubensky ME, Obedin-Maliver J, Lunn MR, Flentje A (2025). US state policies and mental health symptoms among sexual and gender minority adults. JAMA Netw Open.

[4] Miller LR, Grollman EA (2015). The social costs of gender nonconformity for transgender adults: implications for discrimination and health. Sociol Forum (Randolph N J).

[5] Murchison GR, Aguayo-Romero RA, Lett E, Katz-Wise SL, Agénor M, Gordon AR (2023). Transgender/nonbinary young adults’ exposure to cissexism-related social stressors: variation across gender groups. Soc Sci Med.

[6] James SE, Herman JL, Rankin S, Keisling M, Mottet L, Anafi M (2016). The report of the 2015 U.S. Transgender Survey. National Center for Transgender Equality.

[7] Blosnich JR, Marsiglio MC, Dichter ME (2017). Impact of social determinants of health on medical conditions among transgender veterans. Am J Prev Med.

[8] Veale JF (2023). Transgender-related stigma and gender minority stress-related health disparities in Aotearoa New Zealand: hypercholesterolemia, hypertension, myocardial infarction, stroke, diabetes, and general health. Lancet Reg Health West Pac.

[9] Escobar-Viera CG, Whitfield DL, Wessel CB (2018). For better or for worse? A systematic review of the evidence on social media use and depression among lesbian, gay, and bisexual minorities. JMIR Ment Health.

[10] Berger MN, Taba M, Marino JL, Lim MS, Skinner SR (2022). Social media use and health and well-being of lesbian, gay, bisexual, transgender, and queer youth: systematic review. J Med Internet Res.

[11] Vogel EA, Flentje A, Lunn MR (2024). Active social media use and health indicators among sexual and gender minority adults. LGBT Health.

[12] Dowers E, White C, Cook K, Kingsley J (2020). Trans, gender diverse and non-binary adult experiences of social support: a systematic quantitative literature review. Int J Transgend Health.

[13] Eysenbach G (2004). Improving the quality of web surveys: the Checklist for Reporting Results of Internet E-Surveys (CHERRIES). J Med Internet Res.

[14] Selkie E, Adkins V, Masters E, Bajpai A, Shumer D (2020). Transgender adolescents’ uses of social media for social support. J Adolesc Health.

[15] Gordon JD, Whitfield DL, Mammadli T, Escobar-Viera CG (2023). Social support-seeking strategies on social media at the intersection of lesbian, gay, bisexual, transgender, and queer identity, race, and ethnicity: insights for intervention from a qualitative study. JMIR Form Res.

[16] Cipolletta S, Votadoro R, Faccio E (2017). Online support for transgender people: an analysis of forums and social networks. Health Soc Care Community.

[17] Khalaf AM, Alubied AA, Khalaf AM, Rifaey AA (2023). The impact of social media on the mental health of adolescents and young adults: a systematic review. Cureus.

[18] Gerke DR, Step MM, Rünger D (2020). Associations between social support and social media use among young adult cisgender MSM and transgender women living with HIV. Health Promot Pract.

[19] McConnell EA, Clifford A, Korpak AK, Phillips G, Birkett M (2017). Identity, victimization, and support: Facebook experiences and mental health among LGBTQ youth. Comput Human Behav.

[20] Cervantes L, Rizzolo K, Indovina KA (2023). Assessment of a peer support group intervention for undocumented Latinx immigrants with kidney failure. JAMA Netw Open.

[21] Logie CH, Lacombe-Duncan A, Lee-Foon N, Ryan S, Ramsay H (2016). “It’s for us -newcomers, LGBTQ persons, and HIV-positive persons. You feel free to be”: a qualitative study exploring social support group participation among African and Caribbean lesbian, gay, bisexual and transgender newcomers and refugees in Toronto, Canada. BMC Int Health Hum Rights.

[22] Lawlis SM, Butler P, Middleman A (2020). Evaluating transgender youth and parent interest and preferences regarding support groups. Glob Pediatr Health.

[23] Makadon HJ, Mayer K, Potter J, Goldhammer H (2008). Fenway Guide to Lesbian, Gay, Bisexual & Transgender Health.

[24] Poynton TA, DeFouw ER, Morizio LJ (2019). A systematic review of online response rates in four counseling journals. J Couns Dev.

