# Supplementary material for: Survey Evaluation of the Role of Social Media and Social Support for Transgender, Nonbinary, and Intersex People: Observational Study
Source: JMIR Form Res. 2026 Jan 13;10:e79614. doi: 10.2196/79614 (PMC12798915; doi:10.2196/79614)
Supplement: Multimedia Appendix 1 [file formative-v10-e79614-s001.docx]

1. What is your gender identity?
   - transmasculine/transgender man
   - transfeminine/transgender woman
   - Nonbinary
   - Genderfluid
   - other (free text)
2. What is your primary resource for emotional support regarding your gender identity?
   - Spouse, partner, significant other
   - Friends, in person
   - Friends, online
   - Family
   - Social Media
   - Therapy
   - Support group
   - Local organization
   - Other (free text)
3. For the following social media websites, list how many hours per week you each, and what purpose you use each for.

| **Social media site** | **Hours Per Week (whole number)** | **Primary purpose for using site** |
| --- | --- | --- |
| Snapchat |  | Multiple choice answer for each row:   - Interaction with other people, including other transgender or gender diverse people - Social interaction (other) - Consumption of content (ie. watching videos, reading posts, news) - Other (free text) |
| Instagram |  |  |
| Facebook |  |  |
| Linkedin |  |  |
| TikTok |  |  |
| Twitter/X |  |  |
| Tumblr |  |  |
| Discord |  |  |
| Youtube |  |  |
| Reddit |  |  |
| Other (write in, as many as needed) |  |  |
| None |  |  |

1. If you use social media, are the people you interact with online supportive of your gender identity?
   - Yes, all are supportive
   - Yes, some are supportive
   - Most are not supportive
   - None are supportive
2. Do you struggle with any of the following in relation to social media?
   - Overuse
   - Addiction
   - Bullying
   - Self-consciousness
   - Exposure to hate
   - Exposure to transphobia
   - Other (free text)
3. Are you out at your workplace?
   - Yes
   - Yes, to some people but not all
   - No
4. If yes to above, are people in your professional circle supportive of your gender identity?
   - Yes, all are supportive
   - Yes, some are supportive
   - Most are not supportive
   - None are supportive
5. Are you out to your immediate family members?
   - Yes
   - Yes, to some people but not all
   - No
6. If yes to above, are people in your family supportive of your gender identity?
   - Yes, all are supportive
   - Yes, some are supportive
   - Most are not supportive
   - None are supportive
7. How often do you deal with transphobia in your day to day life?
   - Daily
   - Weekly
   - Monthly
   - Never
8. Are your in person friends supportive of your gender identity?
   - Yes, all are supportive
   - Yes, some are supportive
   - Most are not supportive
   - None are supportive
9. Do you currently, or have you ever attended a group session for LGBTQ+ support?
   - Yes, currently attending
   - Yes, but not currently attending
   - No
10. If yes, has this been beneficial? Why? WRITE IN
11. If you answered “Yes, but not currently attending” or “No”, why are you not currently attending a group?
12. If you answered “No”, then are you interested in attending a group that provides support regarding gender identity? Why or why not?
13. Would having a dedicated support group in your life (either in person or virtual) be beneficial to you?
14. If you were to attend a group, would you prefer in-person or virtual?
    - In person
    - Virtual
15. If you were to attend a group, What format would you see being beneficial?
16. Do you regularly see a therapist for any reason?
    - Yes, in the past
    - Yes, currently
    - No
17. Do you have any trans friends?
    - Yes in person
    - Yes online
    - Yes both
    - No
18. Do you have any queer friends?
    - Yes in person
    - Yes online
    - Yes both
    - No

Demographic Information

1. 5-digit zip code
2. Age in years: numerical input
   - 18-24
   - 25-30
   - 30-40
   - 40-50
   - 50-65
   - 65+
3. Please indicate the number of years of school you completed:
   - Did not complete high school
   - Completed high school
   - Vocational training beyond high school
   - Some college (less than 4 years)
   - 4 year college/university degree
   - Graduate or professional degree
4. What best describes your racial heritage:
   - American Indian or Alaska Native
   - Asian
   - Black or African American
   - Native Hawaiian or other Pacific Islander
   - White
   - Other (free text)
   - Decline to respond
5. Please indicate your employment status:
   - Working full-time
   - Working part-time
   - Not currently working
   - Student
   - Retired
6. Please estimate your annual household income from all sources:
   - Less than $25,000
   - $25,000 - $40,000
   - $40,000 - $55,000
   - $55,000 - $70,000
   - $70,000 - $85,000
   - $85,000 - $100,000
   - $100,000 - $150,000
   - $150,000 or more
   - Prefer not to answer
7. Religious affiliation
   - Agnostic
   - Atheist
   - Mormon
   - Catholic
   - Orthodox Christian
   - Other Christian
   - Judiasm
   - Islam
   - Other
   - Prefer not to answer
