# Supplementary material for: Survey Evaluation of the Role of Social Media and Social Support for Transgender, Nonbinary, and Intersex People: Observational Study
Source: JMIR Form Res. 2026 Jan 13;10:e79614. doi: 10.2196/79614 (PMC12798915; doi:10.2196/79614)
Supplement: Checklist 1 [file formative-v10-e79614-s002.docx]

**Checklist for Reporting Results of Internet E-Surveys (CHERRIES)**

| ***Checklist Item*** | ***Explanation*** |
| --- | --- |
| Describe survey design | Volunteers were recruited through an email through the listserv of the LGBTQIA+ employee resource group to review the questionnaire and provide the survey team feedback. The target population was anyone in the LGBTQIA+ population, but this listserv provided the most efficient way to distribution. We had 3 volunteers who provided feedback on the questions and answer choices. The survey was moved from the format of a Word document to Qualtrics platform with review from the Survey Resource Center at the Mayo Clinic. |
| IRB approval | Exempt from IRB approval |
| Informed consent | Informed consent was provided in the form of a recruitment email that emphasized the optional nature of the survey, the benefits of completing the survey, and the fact that no known risks were identified in association with completing the survey. In the recruitment email, participants were told that the survey was short, and that no identifiable information would be collected with the survey. |
| Data protection | No identifiable information was collected for the survey (data was de-identified). |
| Development and testing | Volunteers were recruited through an email through the listserv of the LGBTQIA+ employee resource group to review the questionnaire and provide the survey team feedback. We had 3 volunteers who provided feedback on the questions and answer choices. The survey was moved from the format of a Word document to Qualtrics platform with review from the Survey Resource Center at the Mayo Clinic. |
| Open survey versus closed survey | Closed survey |
| Contact mode | Initial contact was via email provided through the electronic medical record |
| Advertising the survey | Transgender and gender diverse people receiving care from the Transgender and Intersex Specialty Care Clinic (TISCC) at Mayo Clinic Arizona (MCA) were recruited for this study. There were no exclusion criteria among this population. A list of emails was compiled from patients who were seen at the TISCC at MCA between August 16, 2021, until August 14, 2024 using EMR software. |
| Web/E-mail | Web-based survey that was distributed via emailed link with the informed consent and advertising. |
| Context | Transgender and gender diverse people receiving care from the Transgender and Intersex Specialty Care Clinic (TISCC) at Mayo Clinic Arizona (MCA) were recruited for this study. There were no exclusion criteria among this population. A list of emails was compiled from patients who were seen at the TISCC at MCA between August 16, 2021, until August 14, 2024 using EMR software. |
| Mandatory/voluntary | Voluntary |
| Incentives | No incentives were offered |
| Time/Date | September 1, 2024 to October 31, 2024 |
| Randomization of items or questionnaires | Items were not randomized |
| Adaptive questioning | There were conditional questions. |
| Number of Items | 1-3 survey items per page, distributed across 11 pages |
| Number of screens (pages) | 11 |
| Completeness check | Completeness check was not done, but surveys could only be submitted once the last page was reached. |
| Review step | Back button was provided throughout survey |
| Unique site visitor | Emails were sent out with individualized links which only allowed for one response for each individual. |
| View rate (Ratio of unique survey visitors/unique site visitors) | We did not measure view rate |
| Participation rate (Ratio of unique visitors who agreed to participate/unique first survey page visitors) | We did not measure participation rate |
| Completion rate (Ratio of users who finished the survey/users who agreed to participate) | Email list consisted of 236 individuals. 48 responses were received |
| Cookies used | Cookies were not used |
| IP check | IP addresses were not used |
| Log file analysis | No other techniques were used. |
| Registration | Emails were sent out with individualized links which only allowed for one response for each individual. Once the survey assigned to the link was completed the link was not able to be used to answer another survey. |
| Handling of incomplete questionnaires | Only complete questionnaires were analyzed, as participants had to reach the end of the survey to submit. |
| Questionnaires submitted with an atypical timestamp | No cutoff time was used in screening responses. No timestamps were used to evaluate the answers or surveys besides the dates we conducted the survey. |
| Statistical correction | No methods were used such as weight of items or propensity scores were used in the process of analysis. |

This checklist has been modified from Eysenbach G. Improving the quality of Web surveys: the Checklist for Reporting Results of Internet E-Surveys (CHERRIES). J Med Internet Res. 2004 Sep 29;6(3):e34 [erratum in J Med Internet Res. 2012; 14(1): e8.]. Article available at [https://www.jmir.org/2004/3/e34](https://www.jmir.org/2004/3/e34/)/; erratum available <https://www.jmir.org/2012/1/e8/>. Copyright ©Gunther Eysenbach. Originally published in the [Journal of Medical Internet](http://www.jmir.org) Research, 29.9.2004 and 04.01.2012.

This is an open-access article distributed under the terms of the Creative Commons Attribution License (<https://creativecommons.org/licenses/by/2.0/>), which permits unrestricted use, distribution, and reproduction in any medium, provided the original work, first published in the Journal of Medical Internet Research, is properly cited.
